# Experimental Adjustment on Drug Interactions through Intestinal CYP3A Activity in Rat: Impacts of Kampo Medicines Repeat Administered

**DOI:** 10.1093/ecam/nep159

**Published:** 2011-06-05

**Authors:** Natsumi Kinoshita, Yuriko Yamaguchi, Xiao-Long Hou, Kyoko Takahashi, Koichi Takahashi

**Affiliations:** ^1^Department of Pharmaceutics, School of Pharmaceutical Sciences, Mukogawa Women's University, Hyogo 11-68, Koshien, Kyuban-cho, Nishinomiya 663-8179, Japan; ^2^Department of Medicinal Resources, Graduate School of Pharmaceutical Sciences, Osaka University, Osaka, Japan

## Abstract

To provide the information that is necessary for making the proper use of kampo medicines, we have proposed the adequate methodology focused on the following issues: (i) kampo medicines emphasize the effects produced by the combination of herbal drugs rather than the individual effect of any single herb and (ii) Intestinal CYP3A has become a key factor for the bioavailability of orally administrated drugs. In the present study, we investigated both the *in vivo* and *in vitro* effects of Saireito and Hochuekkito (kampo formulas) on CYP3A activities. From our study, oral pre-treatment with Saireito or Hochuekkito did not affect the pharmacokinetics of nifedipine after intravenous administration to rats. When nifedipine was administered to rat intrajejunum, a significant decrease of AUC was showed by pre-treatment with both kampo formulas. Saireito pre-treatment led to 80% decrease in *C*_max_ of nifedipine. Saireito caused significant increases in both protein expression and metabolic activity of CYP3A in intestinal microsome, whereas it had no effect on CYP3A in hepatic microsome. Our result also showed that this affect of Saireito can be gone by wash-out with 1 week. These findings demonstrated that Saireito may induce CYP3A activity of intestine but not of liver in rats. When resources for research are limited, well-designed scientific studies except clinical trials also have many advantages.

## 1. Introduction

The aims for concomitant use of kampo formulas with prescription medicines are: (i) enhancement of medical effects; (ii) reductions of side effects; and (iii) minimizing the dosage of drugs administered [1]. In Japan, kampo medicine was officially integrated into the Japanese healthcare system. Many Japanese medical doctors utilized kampo formulas in their daily practice either as the sole source of therapy or in combination with prescription medicines [2]. Coincidentally, this reversal has occurred following highly publicized problems with herbal medicine safety, reliability and efficacy. Significant harm has been demonstrated by negative interactions of cytochrome P450 (CYP) 3A substrates with the popular herbal medicine, St John's Wort (*Hypericum perforatum* L.) [3, 4].

Kampo medicine is a multi-component system since it is composed of more than one herbal medicine; it is difficult to predict the interaction by accumulating the effect of each component herbal plant. With the development in elucidating the pharmacology of kampo medicines, it is found that each herbal plant plays its indispensable role in a kampo medicine [2]. Saireito and Hochuekkito are major kampo formulas (Table 1) in Japan and they show clinical efficacy in combination with prescription medicines, that is, reducing the dose or side effect of steroids or anticancer drugs [5]. However, because of the absence of data to guide concomitant use of kampo formulas with prescription medicines it is difficult to ensure its robustness.

The safety profiles of multi-medication increasingly require documentation of CYP450 and P-glycoprotein interaction [6]. To provide the information that is necessary for health policy and official recommendations, we have focused on the following issues: (i) kampo formulas emphasize the effects produced by the combination of herbal drugs rather than the individual effect of any single herb; (ii) intestinal CYP3A has become a key factor for the bioavailability of orally administrated drugs. Especially, nifedipine is one of the drugs that have been suggested to undergo significant first-pass metabolism by CYP3A in the intestine [7]. Thus, we tried to evaluate the synthetic effect of kampo as a multi-herb formula and to obtain useful information for providing warning and proper advice to patients in clinical practice.

## 2. Materials and Methods

### 2.1. Materials

Saireito and Hochuekkito extract granules (Table 1) [8] were purchased from Tsumura & Co., Ltd (Tokyo, Japan) (Serial number: 25026892 for Saireito and 23029192 for Hochuekkito). Polyethylene glycol (PEG) 400 was purchased from Nacalai Tesque (Kyoto, Japan). Nifedipine was obtained from Sigma Chemical Co. (St Louis, MO, USA). Oxidized nifedipine was purchased from Sumitomo Chemical Co., Ltd. (Osaka, Japan). Trypsin inhibitor (from soybean) and (*p*-amidinophenyl) methanesulfonyl fluoride hydrochloride (APMSF) were obtained from Wako Pure Chemicals Ltd. (Osaka, Japan). NADP, glucose-6-phosphate and glucose-6-phosphate-dehydrogenase were purchased from Oriental Yeast Co., Ltd. (Tokyo, Japan). All other chemicals available were of the finest reagent grade.

### 2.2. Animals

Male Wistar/ST rats (Japan SLC, Hamamatsu, Japan), weighing 220–290 g, were used in accordance with the Guidelines for Animal Experimentation of Mukogawa Women's University, which are based on the Guidelines for Animal Experimentation of the Japanese Association for Laboratory Animal Science.

### 2.3. Three-Dimensional HPLC Analysis

**Figure 1:**
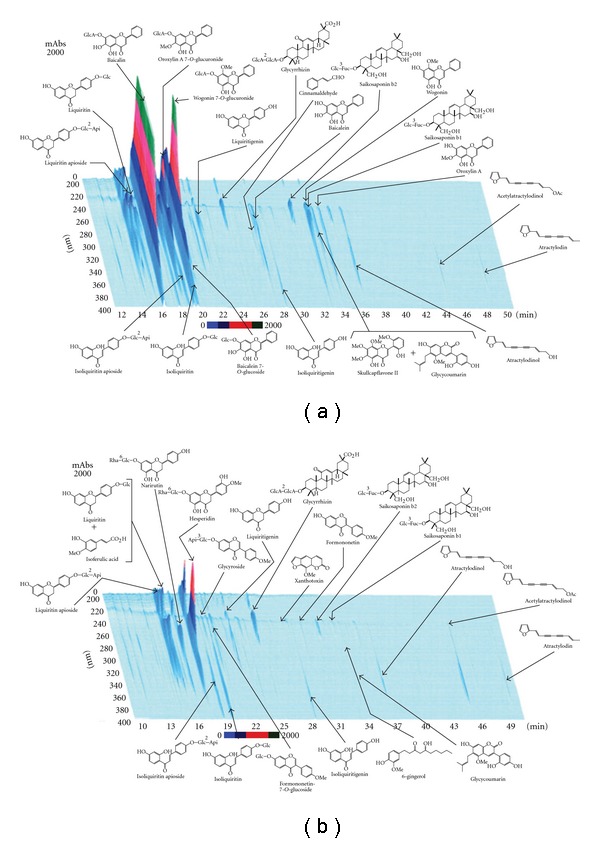
Chemical profile of Saireito (a) and Hochuekkito (b) analyzed by three-dimensional HPLC.

Granules of Saireito and Hochuekkito (1.0 g) were extracted with methanol (20 mL) under ultrasonication for 30 min, and were centrifuged at 1500 g for 5 min. The supernatant was filtrated with a membrane filter (0.45 *μ*m) and then submitted for HPLC analysis (30 *μ*L). HPLC apparatus consisted of a Shimadzu LC 10A (analysis system software: CLASS-M10A ver. 1.64, Tokyo, Japan) equipped with a multiple wavelength detector (UV 200–400 nm) (Shimadzu SPD-M10AVP, diode array detector) and an auto injector (Shimadzu CTO-10AC). HPLC conditions were described as follows: column, ODS (TSK-GEL 80TS, 250 × 4.6 mm i.d., TOSOH, Tokyo, Japan); eluant, (A) 0.05 M AcONH_4_ (pH 3.6) (B) 100% CH_3_CN. A linear gradient of 90% A and 10% B changing over 60 min to 0% A and 100% B was used (and 100% B was continued for 20 min); temperature, 40°C; flow rate, 1.0 mL min^−1^. Figures 1(a) and 1(b) show the chemical profiles of Saireito and Hochuekkito, respectively.

### 2.4. Pre-Treatment of Kampo Medicines

The extract granules of Saireito and Hochuekkito were suspended in solvent of PEG 400/water (1 : 1) as a concentration of 0.3 g mL^−1^ and 0.25 g mL^−1^, respectively, and agitated overnight. Then, rats were orally administered with these suspensions for 7 days (1.5 g kg^−1^ in Saireito and 1.25 g kg^−1^ in Hochuekkito). The control group was administered with solvent.

### 2.5. *In Vivo* Pharmacokinetic Experiment

Intravenous or intrajejunum administration of nifedipine was performed by the method as described earlier [9]. Briefly, following the pre-treatment of kampo medicine, rats were allowed to fast before the experiments for 18–20 h with water freely available. After rats had been anesthetized with ethyl carbamate (1 g kg^−1^), nifedipine dissolved in PEG 400/water (1 : 1) was administered intravenously or intrajejunally (3 mg mL^−1^ kg^−1^). After administration, blood samples (0.5 mL) from the jugular vein were collected periodically. The samples were centrifuged, and the plasma fraction was frozen at –20°C until the HPLC assay of nifedipine.

### 2.6. Nifedipine Measurement of *In Vivo* Experiment

The nifedipine concentrations in rat plasma were measured by HPLC method reported by Takahashi et al. [10]. The plasma samples (200 *μ*L) were deproteinized with acetonitrile (1 mL) and centrifuged. Supernatants (1 mL) were collected and evaporated before being dissolved with mobile phase and 80 *μ*L was injected into the HPLC. The HPLC system consisted of a pump (LC-10ADvp, Shimadzu, Kyoto, Japan), and a UV detector (SPD-10Avp, Shimadzu, Kyoto, Japan) and an integrator (SCL-10Avp, Shimadzu, Kyoto, Japan). The column was TSKgel ODS-80TM column (4.6 × 150 mm: TOSOH Corp., Tokyo, Japan) with the mobile phase of 10 mM KH_2_PO_4_ buffer/acetonitrile (55 : 45) at a flow velocity of 1 mL/min at 40°C. Nifedipine was detected as the absorbance at 350 nm. Nifedipine concentrations for each of the samples were calculated by a standard calibration curve for nifedipine (0.3–150.0 *μ*g mL^−1^). Correlation coefficients were obtained >0.998.

### 2.7. Pharmacokinetic Analysis

The peak plasma concentration (*C*_max_) and the time to reach *C*_max_ (*T*_max_) of nifedipine were determined from the actual data obtained after oral administration. The plasma concentration-time data of intravenous or intrajejunum administration was assessed by non-compartment analysis using MOMENT [11] based on the moment analytic method [12]. Half-life (*t*_1/2_) was calculated by this computer program using the last four point of the concentration. The area under the plasma concentration–time curve from zero to infinity (AUC_0-∞_) and the mean residence time (MRT) from zero to infinity was also calculated by the same computer program. The absolute bioavailability (*F*) of nifedipine after intrajejunum administration (i.j.) was estimated as follows: (AUCi.j. × Di.v.)/(AUCi.v. × Di.j.) × 100.

### 2.8. Preparation of Liver and Intestine Microsomes

The livers of rats were removed, minced, rinsed in ice-cold 0.1 M potassium phosphate buffer (pH 7.5), then homogenized in a Teflon-glass homogenizer immersed in ice using 4 mL of the same buffer per gram of liver. The homogenate was centrifuged at 9000 g for 10 min at 4°C, the pellet was discarded, and the supernatant was centrifuged at 100 000 g for 60 min at 4°C. The resultant supernatant was discarded and the pellet (microsomal fraction) was resuspended in 0.1 M potassium phosphate buffer and stored at –80°C until use. The small intestines of rats were removed and flushed with ice-cold 0.1 M potassium phosphate buffer (pH 7.5) containing 0.1 mM EDTA, 0.5 mM dithiothreitol and 2 mM APMSF. An incision allowed removing the villous layer by scraping with a glass slide. The mucosa was suspended in the same buffer containing 0.5 mg mL^−1^ trypsin inhibitor, and homogenized in a Teflon-glass homogenizer and centrifuged at 10 000 g for 20 min at 4°C. Then the microsomal fraction was obtained by centrifuging the supernatant at 100 000 g for 60 min at 4°C, and the pellets were resuspended in 0.1 M potassium phosphate buffer at –80°C until use. The protein concentrations of these microsomes were determined by the method of Lowry et al. [13] using bovine serum albumin as the standard.

### 2.9. *In Vitro* Nifedipine Metabolic Study by Microsomes

In the microsome experiment, the incubation time was determined to be 10 min since the time-oxidized nifedipine formation rate curve has linear relationship at this time. The amount of oxidized nifedipine was detected and the formation rate was calculated when nifedipine concentration was in the range of 0–200 *μ*M. *K*_m_ and *V*_max_ values were calculated according to the Lineweaver-Burk plots.

The incubation mixture (final volume 0.5 mL) contained liver or intestine microsomes suspension (0.5 mg protein), 5 mM MgCl_2_, 100 mM sodium phosphate buffer pH 7.4 and nifedipine solution. Nifedipine was dissolved in methanol (nifedipine final concentration: 20 *μ*M; methanol final concentration: not >1%, v/v). After pre-incubation of mixture with shaking for 5 min at 37°C, the enzyme reaction was initiated by addition of NADPH generating system consisting of 2 mM NADP^+^, 10 mM gluconse-6-phosphate, 1 U glucose-6-phosphate dehydrogenase. After 10 min of incubation, the reaction was terminated by addition of 2.5 mL acetonitrile. The mixture was centrifuged at 10 000 g for 15 min at 4°C, and 1 mL of supernatant was taken for HPLC measurement of oxidized nifedipine, a metabolite of nifedipine.

### 2.10. Oxidized Nifedipine Measurement of In Vitra Experiment

Supernatants (1 mL) were evaporated before being dissolved with mobile phase (200 *μ*L), and 90 *μ*L was injected into the HPLC. The HPLC system was described earlier. The column was TSKgel ODS-80TM column (4.6 × 150 mm: TOSOH Corp., Tokyo, Japan) with the mobile phase of water/acetonitrile (57 : 43) at a flow velocity of 1 mL min^−1^ at 40°C. Oxidized nifedipine was detected as the absorbance at 254 nm. Oxidized nifedipine concentrations for each of the samples were calculated by a standard calibration curve for oxidized nifedipine (1.0–200.0 *μ*g mL^−1^). Correlation coefficients were obtained greater than 0.998.

### 2.11. *In Vivo* Reversibility Experiment

The pre-treatment of Saireito to the rats was described above. After this treatment, following the cessation of Saireito for 1 week, the *in vivo* pharmacokinetic experiment was examined.

### 2.12. Measurement of CYP3A Protein on Rat Intestine or Liver

The liver and small intestine of rat were homogenized in a Teflon-glass homogenizer immersed in ice using lysis buffer containing 150 mM NaCl, 10 mM Tris (pH 7.4), 1 mM EDTA, 1% Triton X-100, 1% deoxycholic acid and protease inhibitor mixture, followed by centrifugation at 1500 g for 10 min. Protein concentration was determined by the BCA protein assay reagent kit (Pierce, Rockford, USA), and bovine serum albumin was used as a standard. Proteins (12 *μ*g) from total cell lysate were analyzed by SDS-PAGE (10% gel). After blotting, the Immobilon-P membrane (Millipore Corp., Billerica, USA) was blocked with 5% skim milk in PBS with 0.5% Tween 20 at room temperature for 1 h. Immunoblots were incubated at room temperature for 1 h with the primary monoclonal antibody to CYP3A (1 : 1000; Daiichi Pure Chemicals Co., Ltd, Tokyo, Japan). After further washing, the membranes were incubated for 1 h with anti-rabbit IgG horseradish peroxidase conjugate (1 : 3000). The protein was visualized by exposing the membrane to Kodak film for 1–5 min in a dark room. Blots were reprobed with antibody to GAPDH as a loading control. Quantitative analysis of immunoblotted band was performed by computer program (Scion Image, version Beta 4.0.3).

### 2.13. Statistical Analysis

All results were expressed as mean ± SD. The statistical analysis was conducted using Student's or Welch's *t*-test for the differences between two groups, and using one-way ANOVA followed by Bonferroni's multiple analysis for the differences among multiple groups by the computer software “Statcel 2” [14]. A difference of *P* < .05 was considered statistically significant.

## 3. Results

### 3.1. Pharmacokinetic Comparison of Nifedipine Administered Intraveneously or Intrajejunally *In Vivo* Study

**Figure 2:**
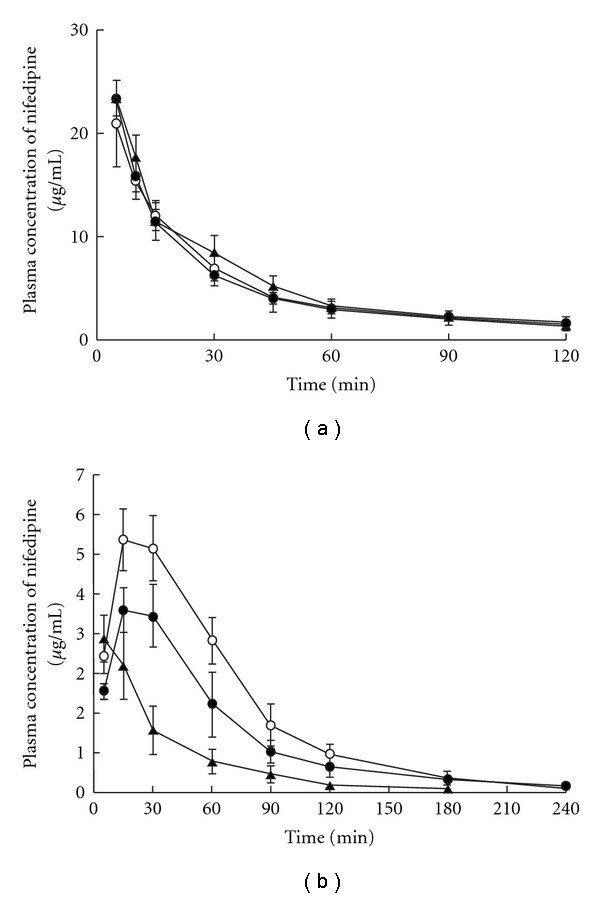
(a) Effects of oral pre-treatment with Saireito and Hochuekkito on the plasma concentration of nifedipine after intravenous administration to rats. (b) Effects of oral pre-treatment with Saireito and Hochuekkito on the plasma concentration of nifedipine after intrajejunum administration to rats. Symbols: control treated with the only solvent (open circle); pre-treatment with Saireito (filled triangle) and Hochuekkito (filled circle) for 1 week, respectively. Each point and vertical bars represent the mean ± SD (*n* = 4).

The effects of repeated administration of kampo formulas on the pharmacokinetics of nifedipine, which is a substrate for CYP3A, were examined *in vivo* and following *in vitro*. When nifedipine was intravenously administered (i.v.) to rats pre-treated with Saireito (1.5 g kg^−1^) or Hochuekkito (1.25 g kg^−1^) for 1 week, their plasma concentration–time profile and pharmacokinetic parameters were unaffected as compared with the control group (Figure 2(a) and Table 2). Whereas, when nifedipine was intrajejunally administrated, Saireito pre-treatment led to a shorter *T*_max_ compared with that of control group, and the *C*_max_ was decreased to ~79%. Thus, AUC_0-∞_ became *∼*43% of that of the control group (Figure 2(b) and Table 2). When rat was treated with Hochuekkito, the AUC_0-∞_ was *∼*71% of that of control group although no difference was observed in *C*_max_ and *T*_max_. In addition, the value of *F* was 56.6% in the control group; however, after treatment with Saireito or Hochuekkito, the values of *F* were 25.6 and 35.3%, respectively.

### 3.2. The Metabolic Activity of Nifedipine for *In Vitro* Study

To investigate the effect of kampo formulas on nifedipine metabolism, we prepared the microsomes of liver and small intestine from rat given Saireito or Hochuekkito orally in advance for 1 week as *in vivo* experiment. Then, microsome was prepared, respectively, to study the affect of kampo medicines on microsome CYP3A.

**Figure 3:**
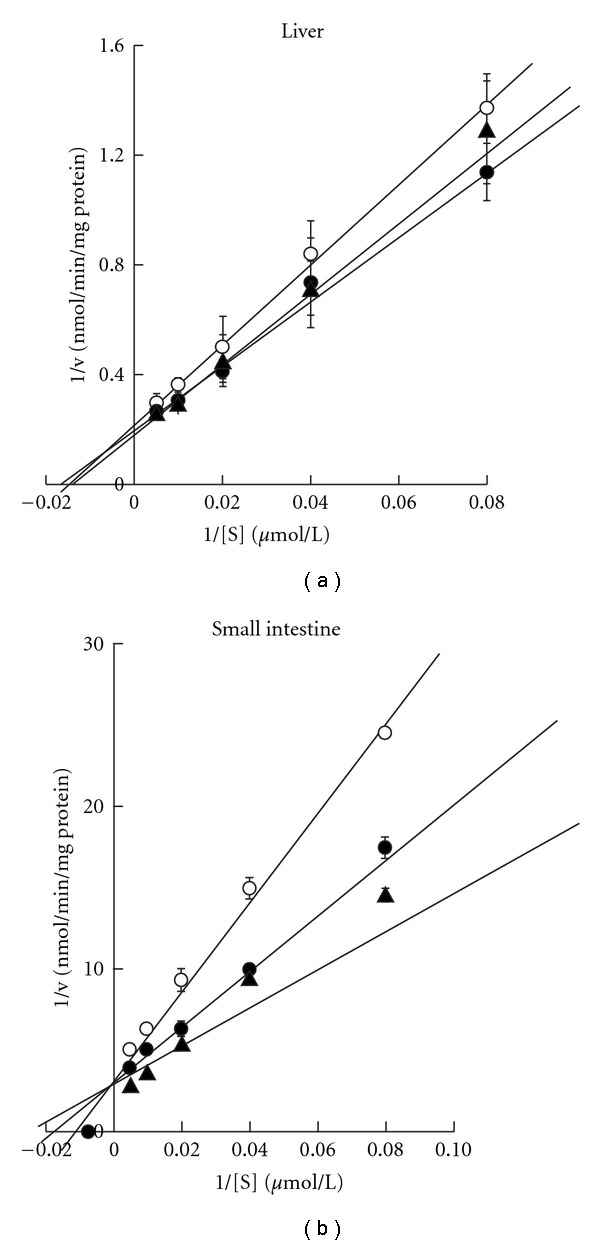
(a) Nifedipine oxidation rates and Lineweaver-Burk plots in rat intestinal microsomes. (b) Nifedipine oxidation rates and Lineweaver–Burk plots in rat hepatic microsomes. Symbols: control treated with the solvent (open circle); pre-treatment with Saireito (filled triangle) and Hochuekkito (filled circle) for 1 week, respectively. Each point and vertical bars represent the mean ± SD (*n* = 4–6).

The concentration profiles were described by Lineweaver-Burk plots and enzyme kinetic parameters were summarized in Table 3. In the liver microsome prepared from repeated pre-treatment of kampo formulas (Saireito and Hochuekkito), the *K*_m_ values and *V*_max_ values among the three groups were similar (Figure 3(a) and Table 3). When small intestine microsome was used, the metabolism of nifedipine was increased (Figure 3(b)). The *V*_max_ values for control group was 0.255 ± 0.020 pmol min^−1^ mg^−1^ protein, and the values for the Saireito and Hochuekkito treated groups were 0.432 ± 0.029 pmol min^−1^ mg^−1^ protein and 0.341 ± 0.048 pmol min^−1^ mg^−1^ protein, respectively. The *K*_m_ values of three groups were not significant as shown in 66.3 ± 6.8 *μ*mol L^−1^ of control, 68.7 ± 8.0 *μ*mol L^−1^ of Saireito and 61.0 ± 5.3 *μ*mol L^−1^ of Hochuekkito. Significant increases in the *V*_max_ values for kampo medicines treated groups were found, and this result confirmed the acceleration of CYP3A activity in small intestine.

### 3.3. The Influence of Kampo Formulas on CYP3A Protein Expression in Liver and Small Intestine

**Figure 4:**
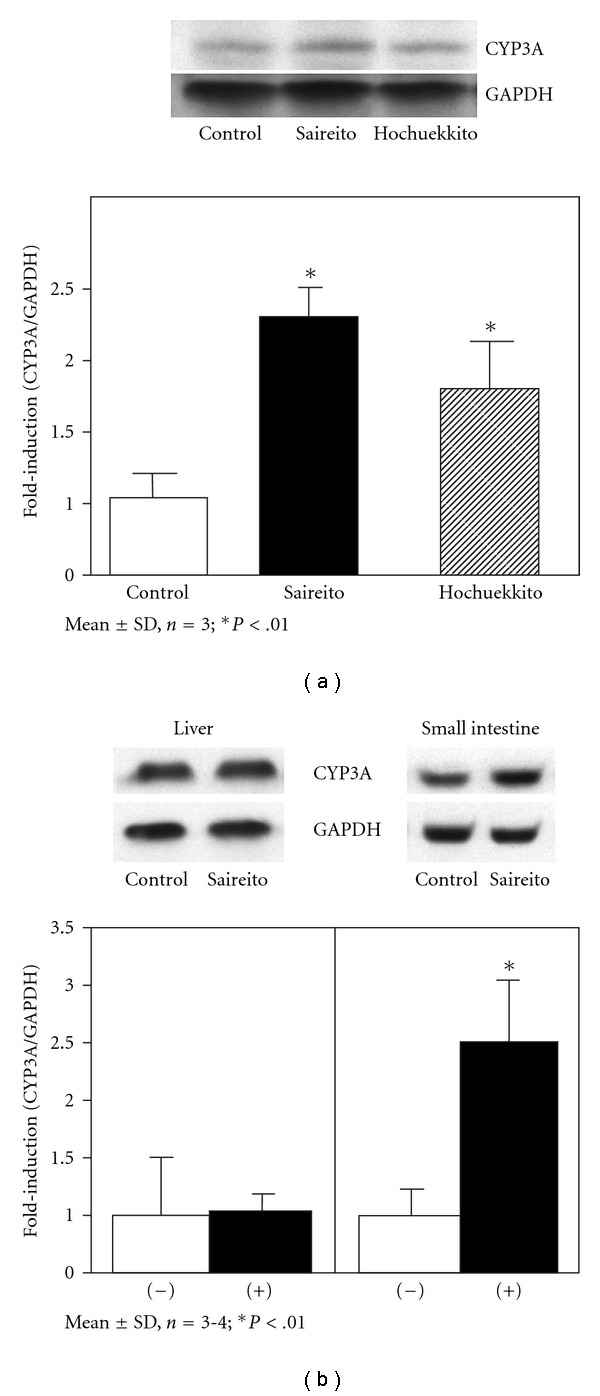
(a) Effects of Saireito and Hochuekkito on CYP3A protein expression levels in rat small intestine. (b) Effects of Saireito on CYP3A protein expression levels in rat small intestine and liver. The band intensities were normalized with that of GAPDH. Results are means ± SD from triplicate experiments. *P < .01 compared with control.

Our *in vivo* results showed that the treatment of Saireito or Hochuekkito would affect the metabolism of nifedipine, and the *in vitro* results indicated an increase in CYP3A activity in the small intestine but not in liver. Western blot was performed to compare the liver and small intestine CYP3A protein expression level in control group to those in the Saireito and Hochuekkito treated groups (Figure 4). In small intestine, Saireito and Hochuekkito treatment increased the CYP3A expression levels by 2.6- and 1.7-fold [Figure 4(a)). As shown in Figure 4(b), after Saireito treatment, although CYP3A expression level in small intestine was significantly induced, no effect was observed on the CYP3A expression level in liver.

### 3.4. The Effect of Saireito Wash-out on the Pharmacokinetics of Nifedipine

**Figure 5:**
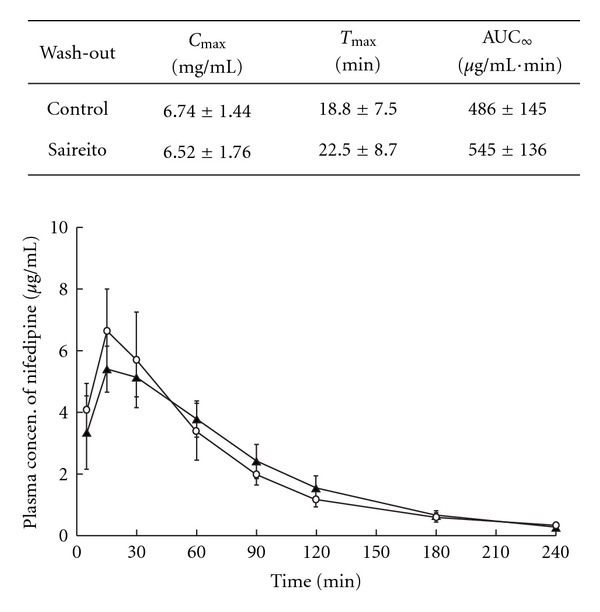
The alteration of nifedipine plasma consentration of intrajejunum administration after Saireito wash-out experiment.

The significant effect of Saireito on nifedipine plasma concentration had been confirmed by both *in vivo* and *in vitro* experiments. Next, after administrated with Saireito for 1 week, rats were further raised for another 1 week without administration of any medicines. The plasma concentration–time profile of nifedipine administrated intrajejunally was measured (Figure 5). After wash-out, similar profiles were resulted between the groups with or without Saireito. The *C*_max_, –*T*_max_, –AUC values in the control group were 6.74 ± 1.44 *μ*g mL^−1^, 18.8 ± 7.5 min, 486 ± 145 *μ*g mL^−1^ min^−1^ and these values in Saireito treated group were 6.52 ± 1.76 *μ*g mL^−1^, 22.5 ± 8.7 min, 545 ± 136 *μ*g^−1^mL^−1^ min^−1^, respectively. The effects of Saireito on the pharmacokinetic parameters of nifedipine were no longer observed 1 week after the withdrawal of Saireito.

## 4. Discussion

In the present study, we investigated both the *in vivo* and *in vitro* effects of Saireito and Hochuekkito on CYP3A activities. The most important findings of this study are the observed interaction for kampo formulas between intestinal CYP3A and a decrease in *F* of nifedipine. We found that the pharmacokinetic parameters of nifedipine after intrajejunum administration were significantly decreased by the continuous ingestion of Saireito and Hochuekkito in advance for 1 week, while the disposition of nifedipine after intravenous administration was not altered. Repeated treatment with kampo formulas increases the rate of metabolism of nifedipine, indicating an increase in CYP3A activity in the intestine. Saireito and Hochuekkito caused significant increases in the metabolic activity of CYP3A in intestinal microsome, whereas it had no effect on CYP3A in hepatic microsome.

Early identification of drugs that interact with kampo medicines and the mechanism involved is important. The first-pass metabolism of nifedipine was reported to be larger in the small intestine than that in liver [7]. Nifedipine has been suggested to be an ideal probe for ascertaining intestinal CYP3A activities. Due to increased intestinal CYP3A, several mechanisms for a decrease in the bioavailability of nifedipine are conceivable. Although the effects of herbal medicines on the function of P-glycoprotein (MDR) may also be important when considering the mechanism of the changes in the pharmacokinetics of orally administered drugs during the absorption process [3], it can be excluded in this case, since nifedipine has been reported not to be a substrate for MDR [15, 16]. Taking into account that the disposition of nifedipine after intravenous administration was not altered by the treatment with kampo formulas, it is likely that the significant decrease in *C*_max_ was brought about mainly by a decrease in *F* particularly due to decreased availability in the process of intestinal mucosal passage. Therefore, the repeated treatment of Saireito would induce the expression and activity of intestinal CYP3A, and further accelerate the metabolism of nifedipine and resulted in a decrease in the bioavailability. In fact, the CYP3A protein appeared to be induced by subchronic treatment with Saireito or Hochuekkito (Figure 3), supporting the idea that formulas are inducer of the intestinal CYP3A isoform *in vivo*. The inductive mechanism of Saireito on CYP3A protein need to be further investigated.

On the other hand, a discrepant report in terms of Saireito (Kanebo Ltd.) on the pharmacokinetic of nifedipine *in vivo* has been published by Ikehata et al. [17]. Although the values of *C*_max_ and AUC for the control groups were higher than those reported by Ikehata, the values of *F* were almost the same. The difference in pharmacokinetics of nifedipine might result from the differences in the experimental method, since in our study rat was anesthetized and nifedipine was intrajejunum administrated. However, in the report by Ikehata, rat was not anesthetized and nifedipine was orally administrated. Moreover, there was no apparent difference between the *in vivo* pharmacokinetics of nifedipine in our study and those in another report by Mohri et al. [18]. Herbal agents are complex mixtures of various phytochemicals, whose absorption and distribution must vary. That may be one of the reasons for the inconsistent results between *in vivo* and *in vitro* studies. Furthermore, this apparent contradiction may arise due to a difference of botanical origin revealed by the scientific taxonomic nomenclature (Japanese Pharmacopeia, 2007), namely *Atractylodes Lancea* Rhizome: Sojutsu (originated in *A. lancea* DE CANDOLLE or *A. chinensis* KOIDZUMI) versus *A. Rhizoma*: Byakujutsu (originated in *A. japonica* KOISZUMI ex KITAMURA or *A. ovata* DE CANDOLLE). The ingredient ratios between both Saireito (by Tsumura Ltd and Kanebo Ltd) are also different. Although both Sojutsu and Byakujutsu are collected in compliance with the Japanese Pharmacopeia, the Saireito recipe depends on each pharmaceutical company. The standards in this Pharmacopeia do not reflect the traditional knowledge on efficacy or safety of the botanical resources. It is important to build modern quality assurance standards for kampo medicines that were based on the standards built up over centuries within traditional health cultures themselves.

We should adopt proper strategies to minimize the negative interactions. It is also notable that the inductive affect by Saireito orally in advance for 7 day was gone. Thus, these models may be used in combination warning and proper advice to patients in clinical practice [19, 20].

Previous *in vitro* studies revealed that schisandra fruit, ephedra herb and cinnamon bark had strong inhibitory effect on microsomal CYP3A activity in rat and human [21, 22]. Makino et al. [23] evaluated the inhibitory effects of the kampo formula (Shoseiryuto) contained above ingredients on rat CYP3A *in vitro* and *in vivo*. Although Shoseiryuto inhibited rat CYP3A activity *in vitro*, it did not significantly affect a plasma concentration profile of nifedipine in rats. Interestingly, clinical studies have also revealed that Shoseiryuto causes no effect on CYP3A4 [24].

Limited literature in negative drug-herb interactions generated inconsistencies and controversies regarding the exact action of these herbs. *In vivo* and *in vitro* screening models will play a major role in identifying possible herb-drug interactions and thus create a platform for clinical studies to emerge [6]. In this study, experimental animals were used for several purposes: (i) to elucidate the affects of repeated orally administrated kampo medicines on CYP3A; (ii) to study the effects of kampo medicine on the kinetics of nifedipine administrated by different ways; (iii) to study the affects of kampo medicine using liver and small intestine microsome; and (iv) to study the reversibility of effects caused by kampo medicine.

## 5. Conclusions

We have demonstrated that subchronic ingestion of Saireito or Hochuekkito may alter the pharmacokinetics of nifedipine. However, since there is marked overlap in the substrates of CYP3A4 and P-gp, the affects of kampo medicines on P-gp should also be investigated *in vivo* to predict changes in the pharmacokinetics of CYP3A substrates. In addition, the effects of more relevant dose used in the present study is approximately 10 times greater than the standard daily dose ingested by humans. Further studies are required before any final conclusion between prescribed medicines and Saireito and Hochuekkito.

## Figures and Tables

**Table 1:** List of components in Saireito and Hochuekkito.

Crude drugs			Ratio crude-drugs component			
Latin name (Japanese name)	Botanical origin	Medicinal part	Saireito		Hochuekkito	
			Amount/day (g)	(%,w/w)	Amount/day (g)	(%,w/w)
*Bupleuri Radix* (Saiko)	*Bupleurum falcatum L. (Umbelliferae)*	Root	7.0	17.5	2.0	8.3
*Pinelliae Tuber* (Hange)	*Pinellia ternata Breitenbach (Araceae)*	Tuber	5.0	12.5	—	—
*Scutellarine Radix* (Ogon)	*Scutellaria baicalensis Georgi (Labiatae)*	Root	3.0	7.5	—	—
*Ginseng Radix* (Ningin)	*Panax ginseng C.A. Meyer, (Araliaceae)*	Root	3.0	7.5	4.0	16.7
*Glycyrrhizae Radix* (Kanzo)	*Glycyrrhiza uralensis Fisher, (Leguminosae)*	Root	2.0	5.0	1.5	6.3
*Zingiberis Rhizoma *(Shokyo)	*Zingiber officinale Roscoe (Zingiberaceae)*	Rhizome	1.0	2.5	0.5	2.1
*Zizyphi Fructus* (Taiso)	*Zizyphus jujuba Miller var. inermis Rehder (Rhamnaceae)*	Fruit	3.0	7.5	2.0	8.3
*Astragali Radix* (Ogi)	*Astragalus membranaceus Bunge, A.mongholicus B. (Leguminosae)*	Root	—	—	4.0	16.7
*Atractylodis Lanceae Rhizoma* (Sojutsu)	*Atractylodes lancea De Candolle, A.chinensis Koidzumi (Compositae)*	Rhizome	3.0	7.5	4.0	16.7
*Angelicae Radix* (Toki)	*Angelica acutiloba Kitagawa, A. a. K. var. sugiyamae Hikino (Umbelliferae)*	Root	—	—	3.0	12.5
*Auranntii Nobilis Pericarpium* (Chimpu)	*Citrus unshiu Markovich, (Rutaceae)*	Peel	—	—	2.0	8.3
*Cimicifugae Rhizoma* (Shoma)	*Cimicifuga foetida L. (Ranunculaceae)*	Rhizome	—	—	1.0	4.2
*Alismatis Rhizome* (Takusha)	*Alisma orientale Juzepczuk (Alismataceae)*	Rhizome	5.0	12.5	—	—
*Polyporus* (Chorei)	*Polyporus umbellatus Fries (Polyporaceae)*	Sclerotium	3.0	7.5	—	—
*Cinnamomi Cortex* (Keihi)	*Cinnamomum cassia Blume (Lauraceae)*	Bark	2.0	5.0	—	—
*Poria* (Bukuryo)	*Poria cocos Wolf (Polyporaceae)*	Sclerotium	3.0	7.5	—	—
		Total: 40.0 g			Total: 24.0 g	

Kampo products, Saireito and Hochuekkito, were provided according to JPXV (http://jpdp.nihs.go.jp/jp15e).

**Table 2:** Effects of oral pre-treatment with Hochuekkito or Saireito on the pharmacokinetic parameters of nifedipine after i.v. or i.j. administration to rats.

Parameter	Control	Hochuekkito	Saireito
i.v.			
AUC_0-∞_ (*μ*g mL^−1^	745 ± 129	807 ± 76	840 ± 91
min^−1^)			
*t*_1/2_ (min)	37.4 ± 3.6	41.4 ± 4.9	35.0 ± 6.7
MRT	37.3 ± 3.6	47.2 ± 8.0	41.4 ± 7.2
i.j.			
*C*_max_ (*μ*g mL^−1^)	5.88 ± 0.61	4.12 ± 0.52*	3.40 ± 0.52*
*T*_max_ (min)	22.5 ± 8.66	30.0 ± 21.2	15 ± 0
AUC_0-∞_ (*μ*g mL^−1^	421 ± 56	285 ± 55*	215 ± 54*
min^−1^)			
*t*_1/2_ (min)	37.1 ± 5.4	41.9 ± 5.3	41.8 ± 2.5
MRT (min)	58.4 ± 8.3	60.0 ± 4.8	54.8 ± 5.1
*F* (%)	56.6 ± 7.6	35.3 ± 6.8*	25.6 ± 6.4*

Each value represents the mean ± SD of 4 or 5 rats.

The nifedipine solution (3 mg/kg) was intravenously (i.v.) or intrajejunal (i.j.) administrated to rats after 7 days pre-treatment with Hochuekkito or Saireito. *P < .05 compared with control.

**Table 3:** Effects of oral pre-treatment with Hochuekkito or Saireito on kinetic parameters of nifedipine oxidation by rat intestinal or hepatic microsomes.

	*K*_m_ (*μ*mol L^−1^)	*V*_max_ (nmol min^−1^ mg^−1^ protein)
Intestine		
Control	66.3 ± 6.8	0.255 ± 0.020
Hochuekkito	61.0 ± 5.3	0.341 ± 0.048*
Saireito	68.7 ± 8.0	0.432 ± 0.029*
Liver		
Control	66.4 ± 7.5	4.50 ± 0.81
Hochuekkito	58.4 ± 2.2	5.12 ± 0.88
Saireito	60.5 ± 8.4	5.05 ± 0.48

Each value represents the mean ± SD of 4 or 5 experiments. The kinetic parameters were calculated from the Linewaver-Burk plots in Figure 3 or Figure 4.

*P < .05 compared with control.
